# Disease-Related Malnutrition and Sarcopenia Predict Worse Outcome in Medical Inpatients: A Cohort Study

**DOI:** 10.3390/nu13092937

**Published:** 2021-08-25

**Authors:** María D. Ballesteros-Pomar, Luisa Mercedes Gajete-Martín, Begoña Pintor-de-la-Maza, Elena González-Arnáiz, Lucía González-Roza, María Pilar García-Pérez, Verónica González-Alonso, María Ascensión García-González, Rocío de Prado-Espinosa, María José Cuevas, Esther Fernández-Perez, José Luis Mostaza-Fernández, Isidoro Cano-Rodríguez

**Affiliations:** 1 Department of Endocrinology and Nutrition, Complejo Asistencial Universitario de León, Gerencia Regional de Salud de Castilla y León (SACYL), Altos de Nava s/n, 24071 León, Spain; lumegm@hotmail.com (L.M.G.-M.); bpintor.asitec@saludcastillayleon.es (B.P.-d.-l.-M.); egonzalezar@saludcastillayleon.es (E.G.-A.); lgonzalezroz@saludcastillayleon.es (L.G.-R.); pgarciapere@saludcastillayleon.es (M.P.G.-P.); icanor@saludcastillayleon.es (I.C.-R.); 2 Department of Internal Medicine, Complejo Asistencial Universitario de León, Gerencia Regional de Salud de Castilla y León (SACYL), Altos de Nava s/n, 24071 León, Spain; vgonzaleza@saludcastillayleon.es (V.G.-A.); agarciagonza@saludcastillayleon.es (M.A.G.-G.); rdeprado@saludcastillayleon.es (R.d.P.-E.); efernandezpe@saludcastillayleon.es (E.F.-P.); jmostaza@saludcastillayleon.es (J.L.M.-F.); 3 Departament of Biomedical Sciences, Institute of Biomedicine, University of León, Campus de Vegazana, 24071 León, Spain; mj.cuevas@unileon.es

**Keywords:** disease-related malnutrition, sarcopenia, GLIM criteria, EWGSOP2, hand grip strength, appendicular lean mass

## Abstract

(1) Background: Both sarcopenia and disease-related malnutrition (DRM) are unfortunately underdiagnosed and undertreated in our Western hospitals, which could lead to worse clinical outcomes. Our objectives included to determine the impact of low muscle mass (MM) and strength, and also DRM and sarcopenia, on clinical outcomes (length of stay, death, readmissions at three months, and quality of life). (2) Methodology: Prospective cohort study in medical inpatients. On admission, MM and hand grip strength (HGS) were assessed. The Global Leadership Initiative on Malnutrition (GLIM) criteria were used to diagnose DRM and EWGSOP2 for sarcopenia. Assessment was repeated after one week and at discharge. Quality of life (EuroQoL-5D), length of stay (LoS), readmissions and mortality are reported. (3) Results: Two hundred medical inpatients, median 76.0 years-old and 68% with high comorbidity. 27.5% met GLIM criteria and 33% sarcopenia on admission, increasing to 38.1% and 52.3% on discharge. Both DRM and sarcopenia were associated with worse QoL. 6.5% died and 32% readmission in 3 months. The odds ratio (OR) of mortality for DRM was 4.36 and for sarcopenia 8.16. Readmissions were significantly associated with sarcopenia (OR = 2.25) but not with DRM. A higher HGS, but not MM, was related to better QoL, less readmissions (OR = 0.947) and lower mortality (OR = 0.848) after adjusting for age, sex, and comorbidity. (4) Conclusions: In medical inpatients, mostly polymorbid, both DRM but specially sarcopenia are associated with poorer quality of life, more readmissions, and higher mortality. Low HGS proved to be a stronger predictor of worse outcomes than MM.

## 1. Introduction

Recently, the main international associations in the field of clinical nutrition have established new criteria for the diagnosis of disease-related malnutrition (DRM), the Global Leadership Initiative on Malnutrition (GLIM) criteria [1] and of sarcopenia, the European Working Group on Sarcopenia in Older People 2 (EWGSOP2) [2], including the assessment of MM and function. The importance of assessing body composition is being increasingly recognized. In addition to its functions in mobility, support, posture, strength, and balance, muscle is currently acknowledged as an organ with important metabolic and homeostatic functions that have been undervalued to date [3]. The loss of muscle mass (MM) associated with disease has been recognized as a clinical condition with potential serious consequences [4]. Muscle mass can be measured by bioimpedance analysis (BIA) in daily clinical practice [3]. Function is also important to monitor and hand grip strength (HGS) has shown to be a good prognostic marker of complications in malnourished patients [5].

Loss of MM by prolonged bedrest decreases functional capacity and increases morbidity and hospital mortality in older adults [6]. Periods of bedrest of only 6–10 days have been reported to lead to significant MM loss [7,8], which is associated with an increase in insulin resistance [9]. The development of sarcopenia during hospitalization has been less studied but also seems to be related to the appearance of complications. Cerri et al. [10] evaluated the prevalence of sarcopenia in 103 hospitalized patients with malnutrition or risk of malnutrition: 21.4% of them presented sarcopenia, which was associated with higher mortality. The international group with the most results in this regard, GLISTEN (Gruppo Lavoro Italiano Sarcopenia—Trattamento e Nutrizione), evaluated the development of sarcopenia in a sample of more than 600 hospitalized elderly people [11,12], 34.7% presented sarcopenia at admission, and among the patients without sarcopenia at hospital admission, 14.7% met the EWGSOP diagnostic criteria for sarcopenia at discharge. This group recently reported [13] that patients with dynapenia, defined by worse results for HGS, had a longer hospital stay. Recently, our group also conducted a pilot study on body composition and muscle strength during hospitalization [14] and observed a decrease in appendicular MM estimated by BIA of 1.2 kg (standard deviation (SD) 1.8, *p* = 0.028) (8.5 kg, SD 3.1 at admission vs. 7.3 kg, SD 3.2 at discharge) and a decrease in HGS of 1.4 kg (SD 5.5). The combination of MM loss with DRM is especially relevant. Studies by Hu et al. [15,16] determined that combined sarcopenia and malnutrition were present in 10.6% of those admitted to a geriatric acute care facility, and the risks of mortality (hazard ratio 4.78) and readmission were high.

Therefore, loss of MM and function are common in hospitalized patients, especially older and polymorbid, and have potential serious adverse effects, but few relevant studies are available. The available evidence supports the relationship of sarcopenia with a worse evolution of hospitalization, but there are doubts about whether it is sarcopenia or malnutrition that is most strongly associated with a poor prognosis. Better knowledge might allow clarification of the risk factors for loss of MM and function and their early detection, so that an adequate nutritional intervention can be considered to prevent deaths or readmissions. Therefore, we investigated the effects of malnutrition and sarcopenia on the clinical prognosis of patients admitted to internal medicine in our hospital, mostly polymorbid patients. The main hypothesis is that low muscle mass (lMM) and function in hospitalized patients and loss during hospital stay are associated to a worse prognosis of the underlying disease. Our objectives were to determine the impact of low MM and strength, and also on prognosis and progress (death, length of stay, readmissions at three months, and quality of life). As secondary objectives, we determined the prevalence of DRM with the GLIM criteria [1] and sarcopenia with the EWGSOP2 criteria [2] in our population and, finally, we assessed the impact of hospitalization on the loss of MM and function.

## 2. Patients and Methods

### 2.1. Type of Study and Ethical Aspects

This prospective cohort study was carried out in adult patients admitted to the Internal Medicine department of the Complejo Asistencial Universitario de León. The study was approved by the clinical research ethics committee of the hospital (number 1936, approval date 26 March 2019) and was carried out in compliance with the ethical and legal standards required for biomedical research according to the Declaration of Helsinki. The purpose of the study was explained to all patients individually, information was provided in writing, and informed consent was requested orally and in writing.

### 2.2. Sample Size Calculation

Our group had previously conducted a pilot study on body composition and muscle strength during hospitalization, and the data obtained were used to estimate the sample size [14]. In addition to MM loss, loss of function seemed more relevant to detect, and we therefore used the HGS data to calculate the needed sample size. To obtain a power of 80% to detect differences contradicting the null hypothesis H_0_: μ_1_ = μ_2_ through a bilateral Student’s t-test for two related samples considering a significance level of 5% and assuming that the initial mean HGS is 21.5 kg, the mean grip strength after hospitalization is 20.1 kg, and the standard deviation of the variable “Difference” is 5.5 kg, 121 patients were required in the study. Considering an expected dropout rate of 15%, 143 patients needed to be recruited. Finally, the sample was expanded to up to 200 patients due to further anticipated losses during the follow-up.

### 2.3. Inclusion and Exclusion Criteria

All patients older than 18 years admitted to the Internal Medicine department during the study period (April−September 2019) with an expected length of stay longer than one week and who were able to provide consent for the study were included. The exclusion criteria were contraindications to BIA (implanted cardiac devices, fever >38 °C or pregnancy), failure to provide signed informed consent, an inability to communicate with the researchers (including severe cognitive impairment), a bedridden status during the three months prior to admission, an isolation requirement due to disease, a terminal prognosis (poor general condition), admission from another service or an intensive care unit (ICU), anasarca or severe edema, and hyperthermia (>38 °C) or hypothermia (<36 °C) during the first 48 h of admission.

### 2.4. Collected and Analyzed Data

An initial visit was held (in the first 48 h of admission), with additional visits at one week after hospitalization and on discharge. To minimize the risk of bias, all clinical assessments were performed by the same researcher and the same scale and same measuring instruments were used for each patient in all the three visits. General data were collected, including the Charlson comorbidity index (CCI) and the degree of functional dependence, by assessing the patients’ activities of daily living according to the Katz index. The Charlson comorbidity index assigns a score of 1, 2, 3 or 6, to 22 different comorbid conditions, depending on the risk of dying associated with each one, and scores are summed to provide a total score to predict one-year mortality. A score higher than 3 is commonly considered as high comorbidity [17]. The Katz index of independence in activities of daily living is an instrument to assess functional status, ranking adequacy of performance in the six functions of bathing, dressing, toileting, transferring, continence, and feeding; patients are scored yes/no for independence in each of the six functions [18]. A score of 6 indicates full function, 4 indicates moderate impairment, and 2 or less indicates severe functional impairment. Anthropometric data were recorded (height, weight, usual weight, the percentage of weight loss, weight loss duration, and calf and arm circumferences). 

Body composition was estimated by electrical bioimpedance (BIA 101^®^ Akern Bioresearch SRL, Pontassieve, Florence, Italy) using electrical values to determine appendicular lean mass (ALM) with the equation proposed by Scafoglieri et al. [19] when analyzing older adults: ALM (kg) = 1.821 + (0.168 ×height^2^/resistance) + (0.132 ×weight) + (0.017×reactance) − (1.931×sex). ALM index (=ALM/height^2^) values below 7 kg/m^2^ in men and 5.5 kg/m^2^ in women are considered low muscle mass (MM) [2]. HGS was measured using a validated DynEx Hand grip^®^ electronic dynamometer [20] (MD System, Inc., Westerville, OH, USA). The patient was seated with the arm adducted at the side, with the elbow flexed to 90° and the forearm in midprone. Hand grip duration was at least 3 s with the dominant hand and the maximum strength of three repeated grips was used as the test score. Values under 27 kg in men and 16 kg in women [2] were considered abnormal. Muscle performance test was assessed using the 30-second chair stand test (recording the number of stands a person can complete in 30 s) [21] and a quality-of-life (QoL) test—the EuroQol 5 Dimension 5 Level (EQ-5D-5L) (https://euroqol.org/ (accessed on 23 August 2021))—was performed after obtaining permission for use. The EQ-5D-5L consists of two pages: the EQ-5D descriptive system and the EQ visual analogue scale (VAS). The descriptive system comprises five dimensions: mobility, self-care, usual activities, pain/discomfort and anxiety/depression. Each dimension has five levels: no problems, slight problems, moderate problems, severe problems and extreme problems. The patient is asked to indicate his/her health state by ticking the box next to the most appropriate statement in each of the five dimensions. This decision results in a one-digit number that expresses the level selected for that dimension. The digits for the five dimensions can be combined into a five-digit number that describes the patient’s health state and this is translated to a score from 5–15 using the calculator provided by EuroQoL. The EQ VAS records the patient’s self-rated health on a vertical visual analogue scale, where the endpoints are labelled ‘the best health you can imagine’ and ‘the worst health you can imagine’. The VAS can be used as a quantitative measure of health outcome that reflect the patient’s own judgement. The following basic analytical data were collected in the first 48 h of admission when available: glucose, C-reactive protein (CRP), and albumin. A 24-h intake record was performed by a dietitian to assess energy and protein intake and analyzed using the software Dietsource 3.0 ^®^ (Nestlé Healthcare Nutrition, Barcelona, Spain).

Nutritional risk screening was performed using the Malnutrition Universal Screening Tool (MUST) as reported by the European Society of Clinical Nutrition and Metabolism [22]. SARC-F was used for screening of sarcopenia [23]. The GLIM criteria for DRM [1] and the EWGSOP2 criteria for sarcopenia [2] were collected at all study visits. To diagnose malnutrition, the GLIM consensus requires that, after risk of malnutrition has been identified by a screening test (i.e., MUST) at least one phenotypic criterion (nonvolitional weight loss, low body mass index, and reduced muscle mass) and one etiologic criterion (reduced food intake or assimilation, and inflammation or disease burden) should be present. The phenotypic criterion defined the severity of malnutrition, according to the cut off points [1]. SARC-F is recommended as a sarcopenia screening tool and comprised of five assessment items: strength, assistance walking, rising from a chair, climbing stairs, and falls. To diagnose sarcopenia, the EWGSOP2 consensus requires that, after risk of sarcopenia has been identified by a screening test (i.e., SARC-F), low muscle function (probable sarcopenia) and mass (confirmed sarcopenia) are present. When assessing MM, the appendicular lean mass estimated by BIA (ALM) or the calf circumference (CC) were used. Low MM by BIA was considered following the EWGSOP2 sarcopenia cut-off points of ASM/height^2^ < 7.0 kg/m^2^ for men and <5.5 kg/m^2^ for women [2]. Calf circumference was measured with the participants seated, at the point of maximum circumference on a plane perpendicular to the long axis of the right calf. The criteria for low CC reported by Santos et al. [24], which are equivalent to two standard deviations below the mean, 29 cm in women and 31 cm in men, were used. One week after admission and/or at discharge, the previous complete assessment was repeated. Data about length of stay (LoS) were recorded during the discharge visit. Patients were offered an appointment in our outpatient clinics 3 months after discharge to assess their clinical progress and to collect data about readmissions and deaths and, in case they did not show up, a telephone call to contact the patient or their relatives or the electronic health record of each patient were used.

### 2.5. Statistical Analysis

All data (tabulation, statistical analysis, and dissemination of the results) were used anonymously. The statistical study was performed using the IBM SPSS Statistics package. Because the variables did not conform to a normal distribution, the data are expressed as the medians (interquartile ranges (IQR)), and nonparametric tests were used to compare medians (the Mann–Whitney U and Kruskal–Wallis tests), while chi-square tests were used for qualitative variables. The associations of LOW MM and HGS and a diagnosis of DRM and/or sarcopenia with length of stay and quality of life (both total and VAS) were assessed by linear regression, including age, sex, and comorbidity in the multivariate model. When deaths and readmissions were assessed, the multivariate method used was logistic regression, also after adjusting for age, sex, and comorbidity

## 3. Results

### 3.1. Characteristics of the Sample

**Figure 1:**
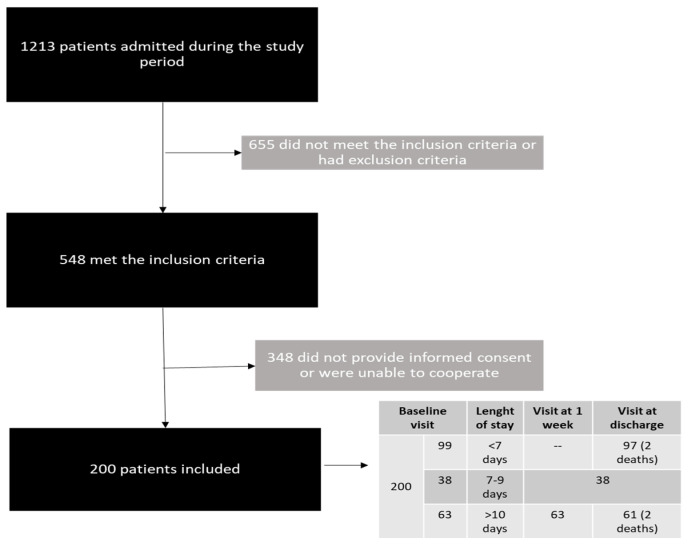
Selection of patients included in the study.

A total of 200 patients who met the inclusion criteria during the study period and agreed to participate were recruited for the study. Figure 1 shows the patient selection process. Of the 200 patients included, 99 had a hospital length of stay of less than 7 days, so the visit considered was at the time of discharge. Thirty-eight had a length of stay between 7 and 9 days, so the visit of the week was also considered the discharge. Finally, 63 patients had a stay of more than 10 days and had one visit at one week and another at discharge (Figure 1). The most frequent reasons for admission were cardiorespiratory (36.5%), digestive (19%), nephrological (9.5%), and oncological diseases (4.5%). Fifty percent of the patients were male, and the median age was 76.0 years (IQR = 20.9). The median CCI was 4 (IQR = 4), and 68% of the patients had a CCI > 3, which is considered indicative of high comorbidity. A total of 18.1% of the patients had moderate disability, and 8% had severe disability according to the Katz index. Nothing per mouth was prescribed for 16% of the patients, a normal or soft/easy-to-chew diet to 80%, and 4% of the patients received a blended diet; only three patients received oral nutritional supplements. The median intake amounts were 30.8 (9.8) Kcal/kg of real body weight and 1.23 (0.44) g/kg of protein for those with a normal diet and 30.2 (16.3) Kcal/kg of real weight and 1.16 (0.68) g/kg of protein for those who had the blended diet. Table 1 shows the baseline clinical characteristics, blood samples data, and body composition data of the patients at admission according to gender.

### 3.2. Anthropometric and Muscle Mass/Function Data

The median initial weight was 68.2 kg (IQR = 20.2), and the median body mass index (BMI) was 26.2 kg/m^2^ (IQR = 6.6), both higher in males (Table 1). 62.8% of the patients had lost weight in the six months prior to admission (median = 2.4%, IQR = 6.2), 49.2% of whom lost more than 5% of body weight, while 20.8% (13% of the total) lost more than 10% of their usual body weight. No significant differences in age, CCI, or sex were observed for those losing more than 5% of weight, although a difference in the Katz index was identified: 0.8 (1.3) in those not losing weight but 1.2 (1.6) if weight loss was higher than 5% (*p* = 0.015). 3% of men and 8.2% of women had a BMI below 20 kg/m^2^, 30% and 40.8% had a BMI of 20–25 kg/m^2^, 42% and 28.6% had a BMI of 25–30 kg/m^2^, and 25% and 22.4% had a BMI greater than 30 kg/m^2^, respectively. Body composition measurements are shown in Table 1. Considering the EWGSOP2 criteria, 90.4% had low MM, without differences according to sex (*p* = 0.386), and 72.9% of the patients had low HGS (70.7% of males and 75% of females, *p* = 0.496) (Table 1).

### 3.3. Screening and Diagnosis of DRM

**Figure 2:**
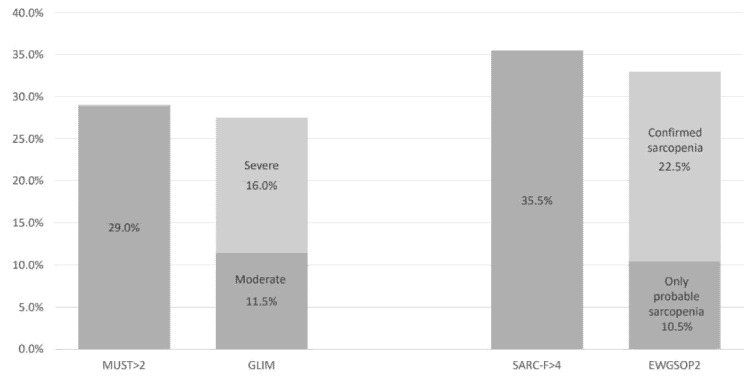
Screening and diagnosis of disease-related malnutrition and sarcopenia at admission. The phenotypic criterion in the GLIM consensus defined the severity of malnutrition, according to the cut-off points [1]. For sarcopenia diagnosed by EWGSOP2, low muscle function implies probable sarcopenia and low muscle mass confirmed sarcopenia [2]. MUST = malnutrition universal screening tool; a score > 2 indicates high risk of malnutrition. SARC-F = sarcopenia screening tool; a score > 4 indicates high risk of sarcopenia.

Figure 2 shows the prevalence of risk of malnutrition according to MUST scores higher than 2 at admission and disease-related malnutrition diagnosed by GLIM. Three patients turned out to be false-positive of the MUST, so the GLIM criteria diagnosed 27.5% of patients on admission and on discharge increased to 38.1%. Considering that all patients met the etiological criteria of “acute disease/injury-related” which motivated their admission, the phenotypic criteria supporting the GLIM diagnosis were weight loss greater than 5% in 67.9% of the patients and greater than 10% in 49.1% of the patients, a BMI lower than 20 kg/m^2^ (or 22 kg/m^2^ in those aged over 70 years) in 25.5% of the patients, a low CC in 64.2%, hand grip strength less than 27 kg in men or 16 kg in women in 74.5% of the patients, and a lower ALM index (less than 7 kg/m^2^ in men and less than 5.5 kg/m^2^ in women) in 100%.

### 3.4. Screening and Diagnosis of Sarcopenia

SARC-F test was higher than 4 in 35.5% of the patients (31% of men and 40% of women, *p* = 0.184). Among those with SARC-F scores > 4, 92.9% had low hand grip strength (90% of men and 95% women), and 75% had chair stand test result below the average for the age; therefore, probable sarcopenia was present in 33% on admission (confirmed sarcopenia in 22.5% of the patients) and 52.3% on discharge, according to the EWGSOP2 criteria (Figure 2).

The combination of DRM according to the GLIM criteria (DRM-GLIM) and sarcopenia occurred in 10.5% of the patients, 17% had only DRM-GLIM without sarcopenia, and 22.5% had only sarcopenia. Table 2 shows the characteristics of the patients based on the diagnoses of DRM and/or sarcopenia. Patients with sarcopenia, alone or in combination with DRM, were older and had greater comorbidity.

### 3.5. Length of Stay, Quality of Life, Readmissions and Mortality and Associations with Low MM and HGS

The median LoS was 7.0 days (IQR = 7.0). The median score on the EQ-5D QoL scale was 6.0 (3.0), and that on the visual analogue scale (VAS) was 50.0 (IQR = 30.0). A total of 6.5% of the patients died and 32% required hospital readmission during the three months of follow-up. 

Low MM measured by either ALM or CC was not significantly related to the LoS or to QoL, or mortality after adjusting for age, sex, and the CCI. However, normal HGS was significantly related to better quality of life, including both total quality of life (Beta = −0.323, *p* = 0.001) and VAS-measured quality of life (Beta = 0.360, *p* < 0.001), and lower risks of readmission (odds ratio (OR) = 0.95, *p* = 0.026) and mortality (OR = 0.85, *p* = 0.014) after adjusting for age, sex, and the CCI, but was not associated with the length of stay (Table 3).

### 3.6. Length of Stay, Quality of Life, Readmissions and Mortality and Associations with DRM or Sarcopenia

After adjusting for age, sex, and the CCI in a multivariate model, a DRM diagnosis with the GLIM criteria was confirmed to be associated with worse quality of life only by the VAS, while a sarcopenia diagnosis with the EWGSOP2 criteria was associated with worse quality of life both globally and as measured by the VAS (Table 3). No association was found with the LoS.

GLIM diagnosis was not significantly associated with a higher readmission rate, but sarcopenia was (42.4% vs. 26.9%, *p* = 0.027). After adjusting for age, sex, and the CCI, the OR for readmission in patients with sarcopenia was 2.25 (95% CI 1.52- 2.99, *p* = 0.03).

Mortality, however, was associated with both the GLIM criteria (12.7% vs. 4.1%, *p* = 0.028) and sarcopenia (16.7% vs. 1.0%, *p* < 0.001). After adjusting for age, sex, and the CCI, DRM was associated with mortality ORs = 4.36 (95% CI 3.11–5.61, *p* = 0.021), and sarcopenia 8.16 (95% CI 6.52–9.79, *p* = 0.012). Comorbid DRM and sarcopenia had an OR of 5.27 (*p* = 0.019). (Table 3). 

### 3.7. Changes in Muscle Mass and Function during Hospitalization

Regarding the evolution of the patients during hospitalization, no significant loss of weight, muscle mass, phase angle measured by BIA, calf circumference, or hand grip strength was observed after one week of admission and only a decrease of ALM of 118 g was observed at discharge (Table 4).

## 4. Discussion

We studied a sample of older adults with high comorbidity (68% with a CCI > 3) who we believe are representative of an increasingly common patient population in Western hospitals. Polymorbid patients are more and more common in our society and have an increased risk of malnutrition, which leads to a worse prognosis during hospitalization; thus, the nutritional approach for such patients is important [25]. 

Our study evaluated the impact of low MM and function, malnutrition, and sarcopenia on clinical and quality of life variables. A diagnosis of DRM or sarcopenia affects a considerable number of older polymorbid patients requiring admission in our study, but its relevance is not limited to prevalence but extends to clinical outcomes. Inflammation related to acute but also chronic disease is both a risk factor for DRM and sarcopenia and also for a worse prognosis. Both diagnoses, DRM and sarcopenia, were associated with poorer quality of life, although the multivariate analysis in the present study did not confirm an association with a longer length of stay. The median stay in our study was short, at only seven days, causing difficulty in showing differences related to nutritional status, such as those found in the PREDyCES study [26]. A higher rate of readmissions was seen only in sarcopenia but not with DRM, reinforcing a better prognostic role for the EWGSOP2 vs. the GLIM criteria. Regarding mortality, sarcopenia criteria were also associated with a higher risk than DRM criteria, corresponding to an eightfold increase in the risk of mortality vs. a fourfold rise with the GLIM criteria. Therefore, using the diagnostic criteria for sarcopenia seems even more relevant in our series than those for DRM. As far as we are aware, there is no study comparing the association of both GLIM and sarcopenia at the same time with QoL, mortality and readmissions in the same population, so we think this is a strong point for our results.

Thus, our data suggest that sarcopenia is associated with a poorer prognosis in a more relevant manner than isolated DRM. The combination of DRM according to the GLIM criteria and probable/confirmed sarcopenia occurred in 10.5% of the patients, confirming previous data reported by other studies in similar populations [15]. Some groups have proposed a malnutrition-sarcopenia syndrome with prognostic implications of higher mortality, with an OR of 4.78 (95% CI, 2.09–10.97) [15], which is confirmed by our data, with an OR of 5.27 (*p* = 0.019).

One striking finding in our population is the high prevalence of low MM, which affected 90.4% of the patients when using the EWGSOP2 criteria, probably due to advanced patient ages and frequent comorbidity/disability in our series. A relationship has been described between low MM and surgical and postoperative complications, with a longer hospital stay, lower physical function, worse quality of life, and lower survival [27]. In our study, since low MM affected almost all patients, we could not assess whether low MM constitutes a poor prognostic factor itself. In addition, in hospitalized patients, frequent clinical practices and treatments (fluids or electrolytes alterations or fluid therapy) may interfere with the performance of BIA, and in our study, this factor caused greater problems for the recruitment and monitoring of patients. Other methods to assess body composition, for example DXA as a reference method, could have been used, but it is not an accessible method at the bedside, as BIA is, and furthermore, the changes in DXA do not allow us to assess differences in the short term. Due to its lower cost and accessibility and absence of irradiation, we decided to use BIA despite recognizing the aforementioned limitations.

However, muscle strength measured by HGS with dynamometry was easier to perform and significantly related to better quality of life and lower risks of readmission and mortality after adjusting for age, sex, and the CCI. Numerous clinical studies have also reported this association between HGS and mortality in different clinical contexts [28,29], which reinforces its role as a prognostic marker. Recently, nutritional supplementation has been reported to improve HGS in patients like ours [30]. In medical inpatients at nutritional risk, HGS has recently showed to provide significant prognostic information about expected mortality and complication risks and helps to identify which patients benefit most from nutritional support [31]. Our study did not include any intervention, and patients did not change their muscle strength during admission, but we can suggest that the data from the literature support the need to propose nutritional treatment in patients with low muscle function measured by HGS.

In Spain, the PREDyCES study found nutritional risk in 23% of hospitalized patients, with patients whose nutritional status worsened during hospitalization having the worst prognosis and the highest health costs [26]. In a previous study, our group found that 27% of patients admitted for medical diseases had an abnormal MUST screening result, confirming a worse evolution in patients with a worsening nutritional status during admission [32]. In the present study, these data were confirmed, with 27.5% of the patients with DRM on admission. The most frequent phenotypic criteria supporting a DRM diagnosis by the GLIM criteria were low ALM estimated by BIA, which affected all patients with nutritional risk in the screening at admission, low function measured by HGS as a surrogate marker, which was observed in 74.5% of the patients, and a low CC, which was observed in 64.2% of the patients. The difficulties of performing BIA in hospitalized patients (the need for equipment and trained personnel, interference from fluid therapy or different circumstances of the patient, etc.), as mentioned, challenge its use as a diagnostic method for DRM. Measuring HGS or calf circumference may be more feasible in the usual clinical context and would not yield a significant false-negative rate in our study. Besides, as previously mentioned, the HGS also has a better predictive capacity.

HGS can also be used to diagnose probable sarcopenia, rendering this parameter even more relevant as a key method in clinical assessments. Sarcopenia was detected in 33% of our patients, which is in line with the rate published by the international group with more related results, GLISTEN (Gruppo Lavoro Italian Sarcopenia—Trattamento e Nutrizione), which detected sarcopenia in 34.7% of elderly patients on admission [11,12].

As noted in previous studies, nutritional status was confirmed to worsen during hospitalization, affecting up to 38% of patients with DRM by the GLIM criteria. These data may reflect the severity of the disease requiring admission but more aptly reflect the insufficient diagnosis and treatment methods for DRM in our hospitals. In our series, only three patients received nutritional supplementation despite the high rate of DRM and sarcopenia. Recent data from the EFFORT study [33] have shown that early diagnosis and adequate nutritional treatment of DRM reduce the risk of mortality by 35% and poor clinical outcomes by 21% and are also cost-effective [34]; therefore, we are obliged to detect and treat DRM in our hospitals. Sarcopenia also worsened during the hospital stay, affecting 52.3% of the patients. Although we still do not have sufficient evidence to determine the best treatment for sarcopenia, some studies suggest an improvement in muscle strength and a decrease in the prevalence of sarcopenia with oral nutritional supplementation [35,36]. More intervention studies will be necessary to confirm these data, but nutritional intervention seems of paramount importance to avoid worse outcomes in relationship with both DRM and sarcopenia.

Our study has some limitations that we must review. First, a significant number of patients did not meet the inclusion criteria or did not want to participate, which we attribute to difficulties in performing BIA and to older polymorbid patients with a poor initial clinical condition. However, we believe that this limitation does not involve a selection bias that affects the validity of our results. The study was carried out in polymorbid and older age patients who, in real life, often present situations similar to those that occurred in our study. The most common causes were that the expected time of admission was less than one week or the poor clinical situation made a near fatal outcome foreseeable, in addition to severe cognitive impairment. In these circumstances, the detection and treatment of DRM and sarcopenia does not offer sufficient clinical benefits. For this reason, we consider that our final sample is representative of a target population in which clinical results can improve with the detection and treatment of DRM and sarcopenia. In addition, the study was conducted in a single department in a single hospital and therefore evaluated only a specific type of patient, mostly older age and polymorbid. Physical activity was not assessed in depth in the study, only the chair stand test was carried out, but not other tests such as the walking speed test which could have added further information about physical performance. Furthermore, the short length of stay did not allow us to determine whether greater losses of muscle mass and function occurred, as reported by other groups [7].

## 5. Conclusions

In medical polymorbid inpatients, both DRM (diagnosed with GLIM criteria) but mostly sarcopenia (with EWGSOP2 criteria) are associated with worse quality of life, more readmissions, and higher mortality. Low muscle strength measured by HGS, but not the amount of muscle mass, was also associated with worse quality of life, a higher readmission rate, and higher mortality. Therefore, we consider that an appropriate approach to DRM and especially to sarcopenia should be reinforced.

## Figures and Tables

**Table 1:** Baseline clinical characteristics, blood samples data and body composition data of the included patients at admission according to gender.

	Male (*n* = 100)	Female (*n* = 100)	*p*
Age (years)	76.9 (15.0)	73.9 (30.0)	0.044
Charlson comorbidity index	5.0 (3.0)	3.5 (5.0)	0.001
Katz index	0 (1)	0 (2)	0.518
Weight (kg)	72.2 (16.2)	64.0 (18.7)	<0.001
BMI (kg/m^2^)	27.0 (5.8)	25.4 (6.6)	0.019
% Weight loss	2.8 (6.2)	1.6 (5.7)	0.254
Arm circumference (cm)	26.0 (4.3)	25.0 (5.1)	0.718
Calf circumference (cm)	32.0 (4.3)	31.0 (5.0)	0.373
Hand grip strength (kg)	21.4 (13.4)	10.5 (8.1)	<0.001
Resistance (Ω)	478.0 (110.5)	567.0 (94.5)	<0.001
Reactance (Ω)	44.0 (13.5)	54.0 (15.0)	<0.001
Phase angle (o)	5.3 (1.3)	5.3 (1.1)	0.611
ALM (kg)	10.4 (2.1)	11.4 (2.6)	0.001
ALMI (kg/m^2^)	3.8 (0.7)	4.6 (0.9)	<0.001
HGS/ALMI (kg/kg/m^2^)	5.4 (3.4)	2.4 (2.1)	<0.001
Blood glucose (mg/dL)	108.0 (38.0)	101.0 (32.0)	0.002
Albumin (g/dL)	3.6 (0.7)	3.7 (0.6)	0.641
C-reactive protein (mg/dL)	56.9 (100.3)	54.8 (127.0)	0.879

Medians (IQR) were compared by the Mann–Whitney U. ALM = appendicular lean mass. ALMI = appendicular lean mass index. HGS = hand grip strength. Charlson comorbidity index ranges 0–33 points; higher than 3 is considered high comorbidity. Katz index ranges 0–6 points; 6 indicates full function, 2 or less indicates severe functional impairment.

**Table 2:** Characteristics of the patients and their progress (length of stay, deaths or readmissions), depending on the diagnoses of DRM by GLIM (DRM-GLIM) and/or sarcopenia.

	Normal	DRM-GLIM	Sarcopenia	DRM-GLIM + Sarcopenia	*p*
*N* (%)	100 (50)	34 (17)	45 (22.5)	21 (10.5)	-
Age (years)	69.4 (28.1)	64.6 (18.5)	81.5 (13.4)	81.0 (11.1)	<0.001
Sex (% men)	53	55.9	48.9	28.6	0.194
CCI	3.0 (4.0)	2.5 (5.0)	5.0 (2.0)	4.0 (4.0)	<0.001
Katz index	0 (1.0)	0 (0)	2.0 (2.0)	2.5 (4.0)	<0.001
Phase angle (o)	5.7 (1.0)	5.4 (1.4)	4.7 (0.9)	4.6 (0.8)	<0.001
HGS (kg)	19.0 (13.1)	18.7 (10.5)	9.2 (6.4)	8.3 (4.4)	<0.001
Length of stay(days)	5.5 (5.0)	7.0 (6.0)	8.5 (7.0)	8.0 (12.0)	0.060
Readmissions (%)	27	26.5	40	47.6	0.151
Deaths (%)	0	5.9	13.3	23.8	<0.001
EuroQol total	6.0 (2.0)	6.0 (1.0)	8.0 (2.0)	8.0 (3.0)	<0.001
VAS	60.0 (30.0)	50.0 (30.0)	50.0 (18.0)	40.0 (28.0)	<0.001

Data expressed as medians (Interquartile range) or % and compared by Kruskal–Wallis test; CCI = Charlson comorbidity index; HGS = handgrip strength; VAS = EuroQol Visual analogue scale (ranges 0–100); EuroQol total range 5–15.

**Table 3:** Relationships of variables related to low muscle mass/function and a diagnosis of DRM and/or sarcopenia with evolution (length of stay, quality of life, deaths or readmissions) after adjustment for age, sex, and comorbidity.

	Total QoL at Admission ^1^	Quality of Life VAS ^1^	Length of Stay ^1^	Readmissions ^2^	Deaths ^2^
ALM	NS	NS	NS	NS	NS
Calf circumference	NS	NS	NS	OR = 0.92 *p* = 0.024	NS
Hand grip strength	Beta = −0.323. *p* = 0.001	Beta = 0.360. *p* < 0.001	NS	OR = 0.95 *p* = 0.026	OR = 0.85 *p* = 0.014
DRM-GLIM	NS	Beta = −0.146. *p* = 0.038	NS	NS	OR = 4.36 *p* = 0.001
Probable + confirmed sarcopenia	Beta = 0.534. *p* < 0.001	Beta = −0.302. *p* < 0.001	NS	OR = 2.25 *p* = 0.030	OR = 8.16 *p* = 0.012
DRM-GLIM + sarcopenia	Beta = 0.263. *p* < 0.001	Beta = −0.179. *p* = 0.015	NS	NS	OR = 5.27 *p* = 0.019

NS = not statistically significant after adjusting for age, sex, and the Charlson comorbidity index; ^1^ linear regression or ^2^ logistic regression. For sarcopenia diagnosed by EWGSOP2, low muscle function implies probable sarcopenia and low muscle mass confirmed sarcopenia [2]. DRM-GLIM=disease related malnutrition with Global Leadership on Malnutrition criteria.

**Table 4:** Changes in muscle mass and function during hospitalization.

	Upon Admission * (*n* = 200)	Changes after One Week (*n* = 101)	Changes at Discharge (*n* = 196)
Initial weight (kg) and changes in weight (%)	68.5 (20.2)	−0.51 (5.0) *p* = 0.488	−0.64 (6.0) *p* = 0.242
Phase angle (^o^)	5.3 (1.3)	5.1 (1.3) *p* = 0.712	5.0 (1.2) *p* = 0.844
Initial ALM and changes (kg)	10.8 (2.4)	−0.091 (0.360) *p* = 0.236	−0.118 (0.490) *p* = 0.039
Hand grip strength and changes (kg)	14.8 (12.9)	−0.5 (4.4) *p* = 0.824	−0.65 (4.9) *p* = 0.364
Calf circumference and changes (cm)	31.0 (5.0)	0.0 (3.0) *p* = 0.537	0.0 (3.0) *p* = 0.767

* Median (IQR). Mann–Whitney U test was performed to compare changes after one week or discharge vs. data upon admission.

## Data Availability

Data supporting reported results available on request.
